# Electrochemically Exfoliated Graphene Quantum Dots Based Biosensor for CD44 Breast Cancer Biomarker

**DOI:** 10.3390/bios12110966

**Published:** 2022-11-03

**Authors:** Neeraj Kumar, Shalu Yadav, Mohd Abubakar Sadique, Raju Khan

**Affiliations:** 1 Industrial Waste Utilization, Nano and Biomaterials, CSIR-Advanced Materials and Processes Research Institute (AMPRI), Hoshangabad Road, Bhopal 462026, India; 2 Academy of Scientific and Innovative Research (AcSIR), Ghaziabad 201002, India

**Keywords:** graphene quantum dots (GQDs), waste dry batteries, electrochemical exfoliation, biosensor, CD44, breast cancer

## Abstract

An innovative electrochemical biosensor based on graphene quantum dots (GQDs) is developed for a simple, rapid, and highly sensitive primary diagnosis of the breast cancer biomarker cluster of differentiation-44 (CD44) antigen. Herein, electrochemical exfoliation of waste dry batteries provides facile, eco-friendly, and cost-effective synthesis of GQDs. Transmission electron microscopy (TEM) analysis reveals that GQDs exhibit spherical shapes with an average diameter of 4.75 nm. Further, electrochemical analysis through cyclic voltammetry (CV) and electrochemical impedance spectroscopy (EIS) reveals that the electrochemical properties of GQDs are suitable for biosensing applications. Subsequently, GQDs have a large electroactive surface area that has been utilized for the immobilization of CD44 antibodies to fabricate the electrochemical biosensor. The electroanalytical performance of GQDs for CD44 biosensing capabilities is studied by differential pulse voltammetry (DPV). The developed electrochemical biosensor has high sensitivity with the lowest detection limit (LOD) of 2.11 fg/mL in the linear range of 0.1 pg/mL to 100.0 ng/mL in phosphate buffer saline (PBS). Further, the linear response of the electrochemical biosensor for CD44 antigen concentration is in the range of 1.0 pg/mL to 100.0 ng/mL with a LOD of 2.71 fg/mL in spiked serum samples. The outcomes suggest that the synthesized GQDs demonstrate promising attributes to be utilized as a viable nanomaterial in biosensing applications.

## 1. Introduction

Cancer is a disease in which cells divide erratically or grow uncontrollably, leading to the emergence of malignancies. Additionally, cell expansion can also move to other organs through veins, causing diseases to affect other organ areas [1]. Breast cancer is the most prevalent cancer in women worldwide, accounting for 0.6 million (6.9%) of all cancer-related deaths. Recent research by Sung et al. indicates that in 2020, 2.3 million (11.7%) cases made it the second biggest cause of cancer deaths worldwide [2]. This malignancy is treatable at the preliminary stage in 70–80% of patients, with an endurance pace of 80% prominent in developed nations [3]. The survival rate in the least developed countries is much lower, below 40%, which is majorly ascribed to the lack of diagnostic techniques at the primary stage of the disease [4].

The detection of breast cancer is majorly dependent on significantly associated biomarkers that resemble the primary growth of cancerous cells. Most commonly known breast cancer biomarkers include cancer antigens (CA); CA 15-3, CA 27-29, protein-6 (CA-6), Human epidermal growth factor receptor 2 (HER2), estrogen receptors (ER), progesterone receptors (PR), circulating tumor cells (CTC), cluster of differentiation-44, 47 (CD44, CD47), mesenchymal-epithelial transition factor receptor (MET), breast cancer genes-1/-2 (BRCA1/2), carcinoembryonic antigen (CEA) [5], and tumor Protein 53 [6]. The CD44 antigen is a potent cancer biomarker that is a multifunctional transmembrane glycoprotein having malignant cellular features such as cell adhesion, activation, migration, and differentiation. CD44 is majorly associated with tumor incursion, evolution, and metastasis [7]. Due to the metastatic cancerous nature of CD44, it is a key biomarker for early diagnosis of breast cancer [8]. However, the biologically relevant detectable range of soluble CD44 in clinical samples is reported to be in the range of ~ 400.0 to 500.0 ng/mL [9].

Traditionally, numerous techniques are utilized for the detection of CD44 antigens, such as the cancer cell, including imaging [10], electrochemiluminescence [11], quartz crystal microbalance [12], gas chromatography (GC) [13], atomic force microscopy [14], high-performance liquid chromatography (HPLC) [15,16], flow cytometry [17,18], spectrophotometry [19,20], thin-layer chromatography (TLC) [21], chemiluminescence [22,23], computed tomography (CT), and magnetic resonance imaging (MRI) [24]. However, these conventional techniques have some disadvantages, such as their expensive, complex, and time-consuming procedures. Hence it is vital to develop a fast, cost-effective, and user-friendly method for the detection of CD44 antigen [25].

Recently, the development of electrochemical biosensors has gained the interest of researchers to detect CD44 antigen effectively due to their numerous advantages, such as a rapid response, cost-effectiveness, simplicity, portability, good accuracy, and high sensitivity as compared to conventional detection tools [26]. Previously, biosensor-based detection of CD44 antigen has been reported in several studies (Table 1). For instance, Zhou et al. developed a hyaluronic acid (HA) with bovine serum albumin (BSA) modified gold nanoparticles (GNPs) (HA-BSA-GNPs) nanocomposite-based electrochemical platform for the determination of CD44 antigen. The developed platform was highly sensitive and specific, with a low detection limit of 128 cells/mL in a linear range from 2.0 × 10^2^ cells/mL to 3.0 × 10^5^ cells/mL [17]. In another study, Ranjan et al. reported an ionic liquid hybrid nanocomposite based-electrochemical immunosensor for highly sensitive detection of CD44 breast cancer biomarkers in clinical samples [27]. Zhao and the research group developed a self-assembled supramolecular nanocomposite-based amplified electrochemical platform for the detection of CD44. The reported platform demonstrated high sensitivity and specificity in a broad linear range from 0.01 ng/mL to 100.0 ng/mL with a low detection limit of 2.17 pg/mL [28]. In another work, they also reported aptamer based-electrochemical impedance sensor for CD44 detection with a LOD of 0.087 ng/mL in a wide linearity range between 0.10 ng/mL to 1000.0 ng/mL [29]. Fan et al. developed a hyaluronic acid (HA) and poly(ethylene glycol) (PEG) hybrid coating on TiO_2_ substrate for sensitive detection of CD44 using the photoelectrochemical (PEC) technique. The reported sensitive detection in a linear range from 0.005 ng/mL to 500.0 ng/mL with an LOD of 0.44 pg/mL [30]. Huang et al. reported a bio-imaging detection of CD44 antigen using fluorescence resonance energy transfer (FRET) from PFEP to fluoresceinamine-hyaluronan (FA-HA) as the fluorescent probe in cancerous cells. The obtained detection limit was 35.0 ng/mL in a linear range from 0.0 ng/mL to 100.0 ng/mL [31]. Qiu and coworkers synthesized an electrochemiluminescence (ECL) probe based on zinc-coadsorbed carbon quantum dots (ZnCQDs) nanocomposites to detect and evaluate CD44 levels in cell lines. The proposed sensor exhibited excellent analytical performance for single MDA-MB-231 cells and MCF-7 cells with a linear range from 1.0 to 18.0 cells and 1.0 to 12.0 cells, respectively. Moreover, the single-cell analysis platform was used for investigating the level of CD44 expression in these two cell lines where MDA-MB-231 cells exhibited 2.8 to 5.2 fold higher CD44 than MCF-7 cells. [32].

Over the years, nanomaterials have been incorporated to increase the effective surface area of the working electrodes and to improve the conductivity of the electrode surface. Further, they also enhance the stability, selectivity, and sensitivity of the electrochemical biosensors. Subsequently, graphene quantum dots (GQDs) have been used in the fabrication of electrochemical biosensors for the detection of various breast cancer biomarkers. GQDs possess a zero-dimensional structure with graphene-like features such as high electrical conductivity and a large surface-to-volume ratio. In addition, GQDs have carboxylic functional groups at the edges, which are vital for the immobilization of bioanalytes [33]. Numerous studies have been reported on GQDs-based electrochemical biosensors for the detection of various breast cancer biomarkers. For instance, Tran et al. developed an ultrasensitive electrochemical biosensor for MCF-7 breast cancer cells by using N-doped GQDs. The developed N-GQDs-based electrochemical platform showed excellent sensitivity and selectivity with a LOD of 1 cell/mL [34]. A thiolated GQDs-based electrochemical immunosensor was reported in another work for the sensitive detection of specific carbohydrate (CA 15-3) breast cancer biomarker and MCF-7 breast cancer cells [35]. To the best of our knowledge, there is no study reported on the electrochemically exfoliated GQDs-based electrochemical biosensing strategy to detect CD44 breast cancer biomarkers.

**Scheme 1:**
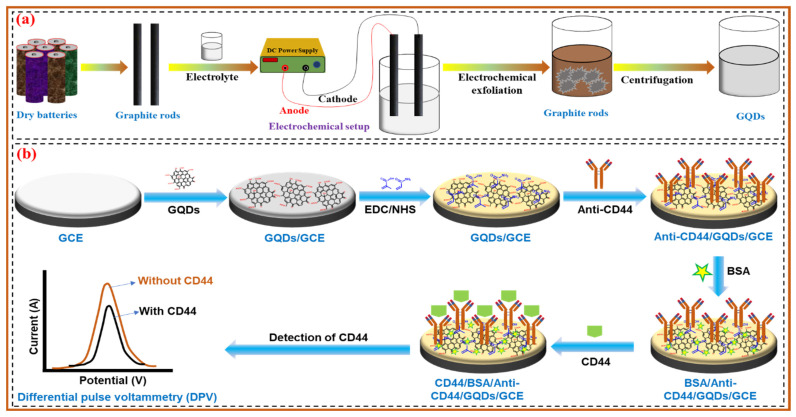
Schematic diagram of the (**a**) synthesis procedure of GQDs and (**b**) sensing mechanism of the fabricated electrochemical biosensor.

In this work, we have developed an electrochemically exfoliated GQDs-based highly sensitive electrochemical biosensor to detect the CD44 breast cancer biomarker for the first time. The use of exfoliated GQDs provides numerous benefits that help in the enhancement of sensor performance. The synthesized GQDs support further advancements in the field of electrochemical biosensors with their significant physio-chemical attributes [36]. The remarkable behavior of GQDs enhances their superior electrochemical properties and their utilization in the development of electrochemical biosensors. The prepared GQDs have an average diameter of 4.75 nm with uniform size. As synthesized GQDs have plenty of carboxylic functionalities and a larger surface that favor the high loading of CD44 antibodies. The fabricated electrochemical biosensor exhibits high sensitivity with the lowest detection limit of 2.11 fg/mL in phosphate buffer saline (PBS) in a wide linearity range between 0.1 pg/mL to 100.0 ng/mL. The proposed platform is also validated in spiked serum samples and provides satisfactory results with a LOD of 2.71 fg/mL in the linear range of 1.0 pg/mL to 100.0 ng/mL. Hence, the developed GQDs-based electrochemical platform would pave the way to detect CD44 and other breast cancer biomarkers in real-life conditions. The fabrication process of GQDs and the sensing mechanism of the fabricated electrochemical biosensor is shown in Scheme 1.

## 2. Materials and Methods

### 2.1. Chemicals

Graphite rods (GRs) from a waste dry battery, sodium hydroxide (NaOH), citric acid (C_6_H_8_O_7_), sulphuric acid (H_2_SO_4_), calcium chloride (CaCl_2_·2H_2_O), dialysis membrane (pore size 2.4 nm), and DC power supply (Powertron) were used to synthesize GQDs. Potassium ferricyanide(III) (K_3_[Fe(CN_6_)]), potassium ferrocyanide(IV) (K_4_[Fe(CN_6_)]), sodium dihydrogen phosphate dihydrate (NaH_2_PO_4_.2H_2_O, 98.0%), disodium hydrogen phosphate (Na_2_HPO_4_), and potassium chloride (KCl), were purchased from SRL Pvt. Ltd., India. CD44 antigen (Product No.-APREST83079), a monoclonal anti-CD44 antibody produced in mouse (Product No.-C7923), bovine serum albumin (BSA), N-(3-Dimethylaminopropyl)-N’-ethyl carbodiimide hydrochloride (EDC), and N-hydroxysulfosuccinimide sodium (NHS) were purchased from Sigma Aldrich, USA. Serum samples were collected from Shivira Pathology, Saket Nagar, Bhopal. All chemicals were used without any refining. All chemical solutions were prepared in ultrapure water (>18 MΩ·cm) from a Millipore Milli-Q water purging framework.

### 2.2. Graphene Quantum Dots (GQDs) Synthesis

The GQDs were synthesized from our earlier work [37]. Briefly, in this approach, the electrodes from two dry batteries were removed. The electrolyte was prepared by mixing 0.1 M citric acid with 1 M NaOH in 40.0 mL Milli-Q water. The electrolyte changed from being a colorless solution to yellow to brown within an hour by gradually applying lower to a higher voltage (2–10 V). The color of the electrolyte solution became deep brown from colorless, indicating the exfoliation of GRs. The solution was further centrifuged and filtered to obtain GQDs.

### 2.3. Modification of the Electrochemical Biosensor

#### 2.3.1. Pre-Treatment of the Working Electrode

Before usage, the glassy carbon electrode (GCE) surface was thoroughly cleaned by following the standard procedure. The electrode was sequentially sonicated and washed for three minutes in 0.1 M H_2_SO_4_ and 95% ethanol. To achieve a shiny mirror surface, all GCEs were polished with first a 0.3 µm α-Al_2_O_3_ slurry and then a 0.05 µm α-Al_2_O_3_ slurry. At last, electrodes were ultrasonicated in distilled water for 5 min and dried before further use.

#### 2.3.2. Fabrication of the Electrochemical Biosensor

At first 5 µL, GQDs solution was drop cast on the GCE surface and kept for drying at ambient temperature for 24 h. Then, 5 µL of freshly made 4: 1:: EDC: NHS (in 10 mM PBS, pH-7.0) were dropped over GQDs modified electrodes to activate the functional groups and then rinsed with Milli-Q water after one hour. After drying at ambient temperature, 5 µL of 20 µg/mL CD44 antibodies (in 10 mM PBS, pH-7.0) were immobilized for 12 h at 4 °C to allow the antibodies to bind with the activated electrode surface. Then, 10 mM PBS, pH-7.0, was used to rinse the CD44 antibody-modified electrode (CD44 antibody/GQDs/GCE). Then after, to prevent non-specific adsorption of analytes onto the CD44 antibody/GQDs/GCE, 1% BSA (in 10 mM PBS, pH-7.0) was used for 40 min. Before experimental tests, the modified electrode (BSA/CD44 antibody/GQDs/GCE) was thoroughly washed with 10 mM PBS, pH-7.0, dried, and kept at 4 °C. The final surface-modified GCE, i.e., BSA/CD44 antibody/GQDs/GCE, was used as the electrochemical probe to detect the CD44 antigen.

## 3. Results and Discussions

### 3.1. Characterization of Graphene Quantum Dots

**Figure 1:**
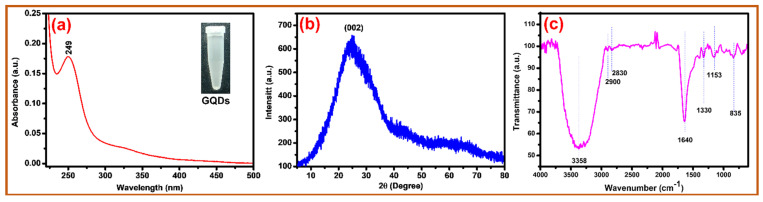
(**a**) UV-vis spectrum, (**b**) XRD spectrum, and (**c**) FTIR spectrum of GQDs.

The synthesized GQDs were characterized for their optical, structural, vibrational, and morphological properties before their utilization in electrochemical biosensing applications. Optical characteristics were observed using a UV-visible spectrophotometer (Thermo Fisher Scientific, Waltham, MA, USA). UV-vis spectrum was recorded in wavelengths of 180 nm to 500 nm. As shown in Figure 1a, the reformation of the π-electron system is compatible with the GQDs having an absorption band at 249 nm, which is attributed to the π-π* transition of ring-structured C = C bonds in the sp^2^ region [38,39,40].

Further, to understand the crystalline nature of the synthesized GQDs, the X-ray diffraction (XRD) pattern was recorded by the MiiFlexII Desktop X-ray Diffractometer (Rigaku Smart Lab Diffractometer). The XRD spectrum, as shown in Figure 1b, was used to identify the crystal phases. A prominent peak observed at $2\theta =24.70^{{}^{\circ}}$ corresponds to the (002) plane of crystalline graphitic structure present in GQDs. The inter-planar spacing (d-spacing) was calculated to be 0.36 nm by the formula nλ=2dsinθ, where n = 1, λ=1.54 Å, $\theta =12.35^{{}^{\circ}}$ at the highest intense peak [41]. The XRD results presented in Figure 1b are in correlation with JCPDS No. 41–1487, which confirms the formation of GQDs [42,43].

The FTIR spectroscopy with ATR Accessory (Model FTIR 4700, JASCO, Tokyo) was used to obtain the FTIR spectra from 4000 cm^−1^ to 600 cm^−1^, as shown in Figure 1c. The FTIR spectrum was used to identify the functional groups present in the GQDs. The bands visible at 3358 cm^−1^ and 1153 cm^−1^ demonstrate intermolecular hydrogen bond (O-H stretching) in GQDs [44]. The graphene sheets (C-H) stretching caused peaks at 2830 cm^−1^ and 2900 cm^−1^. The peak at 1640 cm^−1^ confirms that an sp^2^ carbon structure was recovered in the graphene sheets throughout the reduction process and was predicted to be generated by the (C = C) vibration of the graphene sheets [44,45]. Carboxyl group (C-O) stretching caused the band at around 1330 cm^−1^, while C-C stretching caused another peak at about 835 cm^−1^ [45,46].

**Figure 2:**
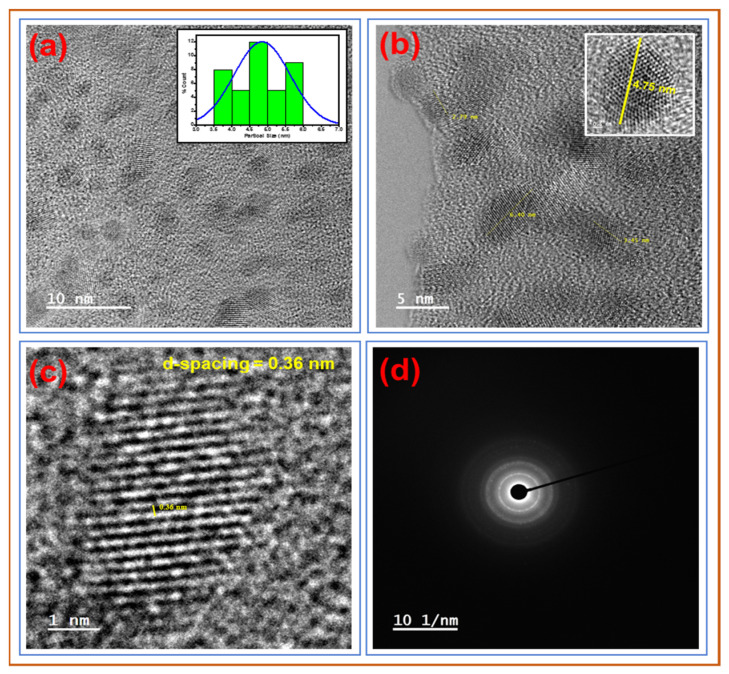
(**a**) TEM image and particle size distribution (inset) (**b**,**c**) HR-TEM images, and (**d**) SAED pattern of GQDs.

A high-resolution transmission electron microscope (HR-TEM) was used to conduct the morphological analysis. The size, shape, and high-resolution images of GQDs were observed using an HR-TEM (Make: JEOL, Model: JEM-F200). The sample (GQDs) was prepared over a carbon-coated copper grid (400 mesh) (Ted-Pella Inc., Redding, CA, USA). TEM analysis was performed for GQDs, as shown in Figure 2. The homogenous distribution of the GQDs is visible in the TEM images at the scale of 10 nm (Figure 2a). The Gaussian fitted size distribution (inset of Figure 2a) of the synthesized GQDs was obtained between 3 nm to 7 nm. The average particle size of GQDs was determined to be 4.75 nm [43]. The different resolutions of the TEM image of the prepared GQDs are shown in Figure 2a,b. The TEM images indicate the synthesized GQDs are spherical and monodisperse. Such particles increase the surface area, which enhances the electrocatalytic properties of prepared GQDs [40]. The HR-TEM image clearly shows the particle size of 4.75 ± 1 nm (inset of Figure 2b) and a d-spacing of 0.36 nm (Figure 2c), which was correlated to the (002) phase of the crystalline graphitic structure. The obtained TEM results were consistent with the calculated XRD data. The semi-crystalline structure of synthesized GQDs was indicated by the presence of a diffuse diffraction ring and spot patterns of the selected area electron diffraction (SAED) image displayed in Figure 2d [37].

### 3.2. Reproducibility of Graphene Quantum Dots

**Figure 3:**
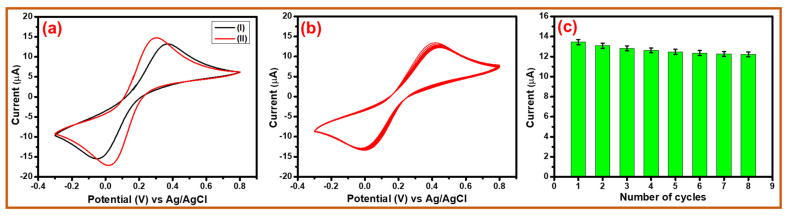
(**a**) Comparative CV response obtained for the bare GCE (I) and GQDs modified electrode (II), (**b**) Consecutive CV scans on single GQDs modified electrode, (**c**) Corresponding bar graph for the reproducibility of GQDs modified electrode.

Before using the synthesized GQDs for biosensing, their electrochemical conductivity and stability studies were carried out via electrochemical techniques. To assess the electrochemical conductivity, the obtained response is compared with bare GCE, and it has been seen that the GQDs have high signal response than bare GCE, as shown in Figure 3a. The electrochemical stability of the GQDs has been evaluated by performing eight consecutive CV scans on the single modified electrode in the potential of 0.3 to 0.8 at the scan rate of 20 mV/s (Figure 3b) and the corresponding change in signal response was evaluated and observed the highly reproducible response up to 90% (Figure 3c) which indicated the potential usability of electrochemically exfoliated GQDs in the fabrication of biosensing platform to detect CD44 efficiently.

### 3.3. Electrochemical Optimization of Electrochemical Biosensor

Electrochemical analysis of surface-modified working electrodes, i.e., GQDs/GCE, CD44 antibody/GQDs/GCE, and BSA/CD44 antibody/GQDs/GCE, have been carried out via different electroanalytical techniques such as Cyclic voltammetry (CV) and electrochemical impedance spectroscopy (EIS). All the electrochemical studies were carried out in 10 mM PBS with KCl (0.1 M) and [Fe(CN_6_)]^3−/4−^ (2 mM) (pH-7.4) as the electrolyte.

#### 3.3.1. Cyclic Voltammetry

CV is widely used to study how chemical species are reduced and oxidized. The study of chemical reactions involving electron transfer, such as catalysis, is significantly aided by CV [47]. The electrochemical biosensor was developed by step-by-step surface modifications of the GCE (i.e., GQDs/GCE, CD44 antibody/GQDs/GCE, and BSA/CD44 antibody/GQDs/GCE) electrode, which were characterized by CV in the potential window of −0.3 V to 0.8 V with a scan rate of 20 mV/s, in the presence of PBS. As shown in 4a, the formation of CD44 antibody/GQDs/GCE after the immobilization of the CD44 antibodies onto the GQDs/GCE had a low current response, indicating that the CD44 antibody was successfully immobilized on the GQDs/GCE electrode surface. The formation of the biolayer after the immobilization of CD44 antibodies at the GQD/GCE hindered electron transfer due to the insulating behavior of bio-species [27]. Additionally, the immobilization of BSA as a blockage agent to produce BSA/CD44 antibody/GQDs/GCE reduced the current, confirming the blocking of non-specific sites on the electrode surface. After each modification step, the peak current value of the working electrodes GCE, GQDs/GCE, CD44 antibody/GQDs/GCE, and BSA/CD44 antibody/GQDs/GCE was 13.21 μA, 14.75 μA, 10.92 μA, and 10.11 μA, respectively. The highest peak current observed for GQDs/GCE was attributed to the highly conducting nature of GQDs.

Using the Brown–Anson method and the equation I_p_ = n^2^F^2^ I^*^AV/4RT, where I* is the surface concentration of the GCE (mol/cm^2^), A is the surface area of the electrode (0.07 cm^2^), V is the scan rate (20 × 10^−3^ V/s), R is the gas constant (8.314 J/mol K), and T is the absolute temperature (298 K), where n is the number of electrons transferred (n = 1), and F is the Faraday constant (F = 96,485.33 C/mol), the surface concentration of the GCE, GQDs/GCE, CD44 antibody/GQDs/GCE, and BSA/CD44 antibody/GQDs/GCE electrodes have been determined [48]. The surface concentration of the GQDs/GCE (112.21 × 10^−10^ mol/cm^2^) was higher than that of the GCE (100.47 × 10^−10^ mol/cm^2^), CD44 antibody/GQDs/GCE (83.05 × 10^−10^ mol/cm^2^), and BSA/CD44 antibody/GQDs/GCE (76.94 × 10^−10^ mol/cm^2^). The significant surface concentration of GQDs/GCE enabled more binding sites for enhanced electrochemical biosensor performance.

#### 3.3.2. Redox Behavior Study

**Figure 4:**
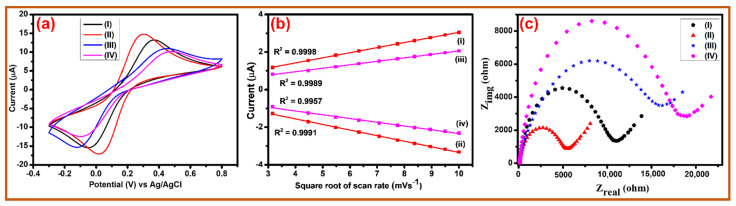
Comparison of the modified electrodes in 10 mM PBS with KCl (0.1 M) and [Fe(CN_6_)]^3−/4−^ (2 mM) (pH 7.4) via (**a**) CV, (**b**) scan rate study, (i, ii) Ipa and Ipc of GQDs, (iii, iv) Ipa and Ipc of the BSA/CD44 antibody/GQDs/GCE, and (**c**) EIS graph for the (I) GCE, (II) GQDs/GCE, (III) CD44 antibody/GQDs/GCE, (IV) BSA/CD44 antibody/GQDs/GCE.

The influence of the scan rate (υ) has been investigated to better understand the reactions on the electrode corresponding to anodic and cathodic peak currents. The scan rate controls the redox and diffusion control electron transfer mechanism of the modified electrodes. A linear plot of CV was obtained (Figure 4b) to compare the CV for GQDs/GCE and BSA/CD44 antibody/GQDs/GCE at the potential windows of −0.3 V to +0.8 V in the variable scan rate range from 10 mV/s to 100 mV/s with the interval of 10 mV/s. Results showed that as the scan rate was increased, the anodic peak current (Ipa) increased while the cathodic peak current (Ipc) decreased linearly, indicating an excellent redox nature. The increasing scan rate reduced the diffusion layer of the electrode resulting in higher current responses [47].

A linear relation of the Ipa and Ipc vs. υ^1/2^ and Epa and Epc vs. υ^1/2^ of the GQDs/GCE and BSA/CD44 antibody/GQDs/GCE is given below and shown in Figure 4b:

Ipa (GQDs/GCE): 0.2717 × υ^1/2^ + 0.3330, R^2^ = 0.9998
Ipc (GQDs/GCE): −0.2978× υ^1/2^ − 0.3657, R^2^ = 0.9991
Ipa (BSA/CD44 antibody/GQDs/GCE): 0.1847 × υ^1/2^ + 0.2124, R^2^ = 0.9989
Ipc (BSA/CD44 antibody/GQDs/GCE): −0.2002 × υ^1/2^ − 0.3366, R^2^ = 0.9957

The values of the regression coefficient (R^2^) from the linear plot support the dependence of the scan rate on the redox nature of the modified electrodes.

#### 3.3.3. Electrochemical Impedance Spectroscopy

EIS studies were conducted on the sequentially assembled electrodes for fabrication of the electrochemical biosensor from the viewpoint of resistance change at the modified electrode surface. In the Nyquist plots, as shown in Figure 4c, the charge-transfer resistance (R_CT_) is equivalent to the diameter of the semicircle. Further, the surface-modified GCE was studied to validate the efficient capture of CD44 antibodies onto the GQDs/GCE. The R_CT_ values for the GCE, GQDs/GCE CD44 antibody/GQDs/GCE, and BSA/CD44 antibody/GQDs/GCE had been obtained as 110.84. × 10^2^ Ω, 55.32 × 10^2^ Ω, 162.19 × 10^2^ Ω, and 189.61 × 10^2^ Ω, respectively. The low R_CT_ value of GQDs/GCE suggested that it had the highest electroconductivity. The corresponding R_CT_ values of CD44 antibody/GQDs/GCE and BSA/CD44 antibody/GQDs/GCE indicated an increment in impedance when compared to GQDs/GCE. The corresponding increment in impedance was due to the successful immobilization of CD44 antibodies and blocking by BSA on the electrode surface, respectively. The successful fabrication of the electrochemical biosensor has been verified through electrochemical techniques.

### 3.4. Performance of Electrochemical Biosensor

The CD44 antigen was detected by a sensitive analytical technique: differential pulse voltammetry (DPV), using the BSA/CD44 antibody/GQDs/GCE electrochemical probe.

#### 3.4.1. Detection of CD 44 Antigen in PBS

**Figure 5:**
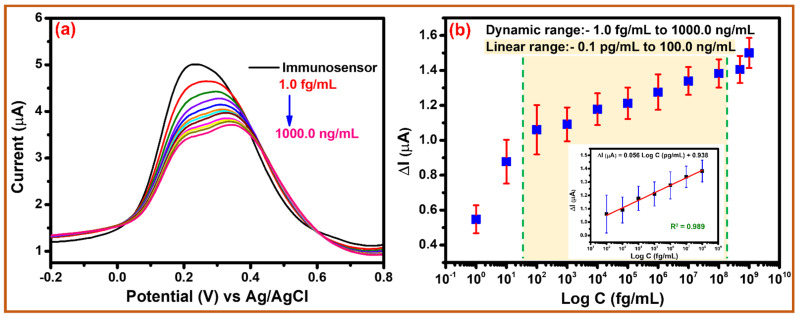
(**a**) Detection of CD44 antigen in PBS (**b**) corresponding calibration graph (Conc. 1.0 fg/mL to 1000.0 ng/mL), inset linear regression curve of the biosensor in PBS used for the calculation of LOD in the linear range from 0.1 pg/mL to 100.0 ng/mL.

DPV is an ultrasensitive analytical approach that can measure only faradaic current with negligible capacitive current, which indicates the improvement of the sensitivity. The linear proportionality of signal response in a DPV curve with the concentration of analyte enables direct quantitative detection of target analytes [49]. Here, the sensing of the CD44 antigen was performed in PBS in a wide dynamic concentration range from 1.0 fg/mL to 1000.0 ng/mL (1.0 fg/mL, 0.01 pg/mL, 0.1 pg/mL, 1.0 pg/mL, 0.01 ng/mL, 0.1 ng/mL, 1.0 ng/mL, 10.0 ng/mL, 100.0 ng/mL, 500.0 ng/mL, and 1000.0 ng/mL). However, the biosensor performed linearly in the concentration range from 0.1 pg/mL to 100.0 ng/mL (0.1 pg/mL, 1.0 pg/mL, 0.01 ng/mL, 0.1 ng/mL, 1.0 ng/mL, 10.0 ng/mL, and 100.0 ng/mL). The respective peak currents were taken to analyze the relationship between the current response and the concentration of the target analyte. Results showed a gradual decrease in the peak current with the increase in concentrations of CD44 antigen. The binding of CD44 antigen with CD44 antibody is responsible for restricting the flow of electrons in the electrolyte, which results in reduced peak current. The limit of detection (LOD) and limit of quantification (LOQ) of the fabricated electrochemical biosensor were calculated using the formulae LOD = 3.3σ/S and LOQ = 10σ/S, where σ is the standard deviation and S is the slope of the calibration graph [47]. The LOD and LOQ in PBS were calculated to be 2.11 fg/mL and 6.40 fg/mL, respectively, with high sensitivity of 2.12 µA cm^−2^/(fg/mL). Figure 5a,b displayed a DPV response curve for the detection of CD44 antigen from 1.0 fg/mL to 1000.0 ng/mL and the corresponding calibration graph, respectively. The inset of Figure 5b shows the linear regression curve of the biosensor in PBS used for the calculation of LOD in the linear range from 0.1 pg/mL to 100.0 ng/mL.

#### 3.4.2. Detection of CD 44 Antigen in Spiked Serum Samples

**Figure 6:**
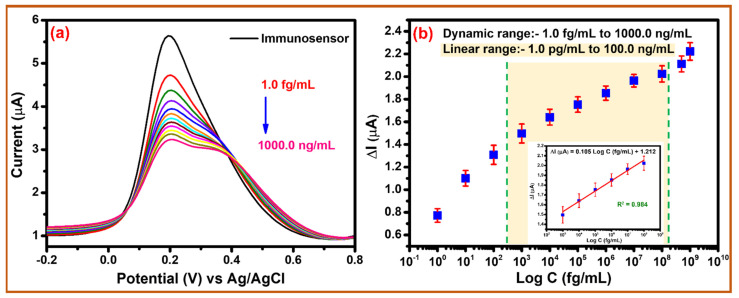
(**a**) Detection of CD44 antigen in spiked serum samples (**b**) corresponding calibration graph (Conc. 1.0 fg/mL to 1000.0 ng/mL), inset linear regression curve of the biosensor in serum used for the calculation of LOD in the linear range from 1.0 pg/mL to 100.0 ng/mL.

To validate the performance of the fabricated electrochemical biosensor, the analytical detection of the CD44 antigen was carried out in spiked serum samples. Similar to the PBS electrolyte, the serum samples were diluted in a 1:10 ratio, and then various concentrations of CD44 antigen were spiked in the diluted serum samples for quantitative detection through DPV. As shown in Figure 6a, the increase in the concentration of CD44 antigen showed a dependent decrease in the current response in the linear range from 1.0 pg/mL to 100.0 ng/mL. The current response was dependent on the concentration of CD44 antigen, suggesting satisfactory binding of the target analyte onto the surface of the electrochemical probe. The consistency of results in diluted serum samples similar to that in PBS evidenced the effective working of the fabricated electrochemical biosensor in real samples as well. The respective peak current for different concentrations was obtained from the DPV curve (Figure 6a), and a corresponding calibration curve was plotted for analytical and statistical studies, as shown in Figure 6b. The inset of Figure 6b shows the linear regression curve of the biosensor in serum used for the calculation of LOD in the linear range from 1.0 pg/mL to 100.0 ng/mL. The LOD and LOQ values of the electrochemical biosensor for spiked serum samples were 2.71 fg/mL and 8.21 fg/mL, respectively. Moreover, the sensitivity of the electrochemical biosensor in spiked serum samples was calculated to be 3.52 µA cm^−2^/(fg/mL).

### 3.5. Reproducibility and Selectivity and Studies

**Figure 7:**
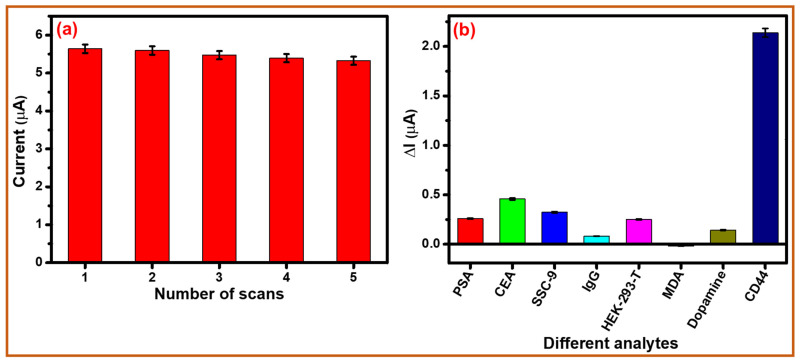
(**a**) Reproducibility analysis of fabricated immunosensor (**b**) selectivity study of the fabricated biosensor in different interfering analytes (Conc. 50.0 pg/mL).

The electrochemical stability of the biosensor was evaluated by performing five consecutive DPV scans, and its corresponding bar graph is shown in Figure 7a. The signal response revealed a very low relative standard deviation (RSD = 5.55%) for the Ip of the PBS. The obtained results are attributed to the high electrochemical stability of fabricated biosensors.

The selectivity of the fabricated biosensor was examined by investigating different interfering analytes, including prostate-specific antigen (PSA), CEA, squamous cell carcinoma (SCC-9) cells, immunoglobulin G (IgG), malondialdehyde (MDA), human embryonic kidney 293 (HEK-293-T) cells, and dopamine having a concentration of 50.0 pg/mL through DPV. The corresponding bar diagram is illustrated in Figure 7b. The results showed that no significant response had been observed for the interfering analyte, while a significant signal response had been observed for the CD44 (50.0 pg/mL) detection. Hence the biosensor is highly selective for the detection of CD44. The studies suggest the potential usage of exfoliated GQDs in next-generation point-of-care diagnostics.

## 4. Conclusions

To summarize, the GQDs were synthesized through a simple, cost-effective, and eco-friendly electrochemical exfoliation method. The electrochemically exfoliated GQDs possessed uniform and small sizes with an average diameter of 4.75 nm, high conductivity, and several oxygen functionalities. In addition, they exhibit good water dispersibility, pH stability (pH = 7.4), and excellent biocompatibility with high electrochemical stability. The synthesized GQDs having peculiar properties support their utilization in the fabrication of electrochemical biosensing platforms.

Further, the GCE has been modified with synthesized GQDs via physisorption, and eight consecutive CV scans were performed to evaluate the electrochemical stability. The results revealed that the GQDs were highly stable, with 90% reproducible response values. The obtained characterization results of GQDs suggest a stable and electroactive nanomaterial exhibiting promising sensing capabilities. Moreover, the high surface concentration of GQDs provides abundant binding sites for biorecognition elements. The GQDs modified electrode served as the anti-CD44 immobilization matrix, at which CD44 could bind effectively and permit the label-free quantification in PBS as well as in diluted human serum over a broad dynamic range between 1.0 fg/mL to 1000.0 ng/mL. Subsequently, the GQDs are used for the fabrication of an electrochemical biosensor as the sensing probe for detecting the CD44 antigen with high sensitivity of 2.12 µA cm^−2^/(fg/mL). The ultra-low detection limit of 2.11 fg/mL and linearity of 0.1 pg/mL to 100.0 ng/mL in PBS approve its applicability in clinical samples.

In addition, the results obtained in spiked serum samples validate the performance of the fabricated electrochemical biosensor for sensitive detection of CD44 antigen. The biosensor could detect CD44 without any significant interference from different interfering analytes such as PSA, SSC-9, HEK-293-T, IgG, dopamine, and CEA. The results obtained from the electrochemical studies suggest a successful development of an efficient electrochemical biosensor for the selective, sensitive, and rapid detection of CD44 breast cancer biomarkers. Moreover, the fabricated electrochemical biosensor has the potential to be translated into point-of-care testing platforms for practical clinical tests.

## Figures and Tables

**Table 1:** Reported biosensors for CD44 breast cancer biomarker detection.

S. No.	Sensing Probe	Medium	Technique	Linearity	LOD	Citation
1.	HA-BSA-GNPs/GCEs	PBS	EIS	2.0 × 10^2^ cells/mL to 3.0 × 10^5^ cells/mL	128 cells/mL	[17]
2.	FF-AuNPs	Serum	LSV	0.01 ng/mL to 100.0 ng/mL	2.17 pg/mL	[28]
3.	Au-Aptamer	Serum	EIS	0.10 ng/mL to 1000.0 ng/mL	0.087 ng/mL	[29]
4.	ITO/TiO_2_/PDA−HA−PEG	Serum	PEC	0.005 ng/mL to 500.0 ng/mL	0.44 pg/mL	[30]
5.	PFEP/FA-HA	Serum	Fluorescence	0.0 ng/mL to 100.0 ng/mL	35.0 ng/mL	[31]
6.	ZnCQDs	MDA-MB-231 cells	ECL	1 cell to 18 cells	-	[32]
7.	Exfoliated GQDs	PBS, Serum	DPV	0.1 pg/mL to 100.0 ng/mL 1.0 pg/mL to 100.0 ng/mL	2.11 fg/mL, 2.71 fg/mL	**This work**

## Data Availability

Not applicable.
